# The effect of acceptance and commitment therapy on insomnia and sleep quality: A systematic review

**DOI:** 10.1186/s12883-020-01883-1

**Published:** 2020-08-13

**Authors:** Nader Salari, Habibolah Khazaie, Amin Hosseinian-Far, Behnam Khaledi-Paveh, Hooman Ghasemi, Masoud Mohammadi, Shamarina Shohaimi

**Affiliations:** 1 Department of Biostatistics, School of Health, Kermanshah University of Medical Sciences, Kermanshah, Iran; 2 Sleep Disorders Research Center, Kermanshah University of Medical Sciences, Kermanshah, Iran; 3 Department of Business Systems & Operations, University of Northampton, Northampton, UK; 4 Department of Nursing, School of Nursing and Midwifery, Kermanshah University of Medical Sciences, Kermanshah, Iran; 5 Department of Biology, Faculty of Science, University Putra Malaysia, Serdang, Selangor Malaysia

**Keywords:** ACT, Behavioral therapy, Insomnia, Sleep quality

## Abstract

**Background:**

Acceptance and Commitment Therapy (ACT), as a type of behavioral therapy, attempts to respond to changes in people’s performance and their relationship to events. ACT can affect sleep quality by providing techniques to enhance the flexibility of patients’ thoughts, yet maintaining mindfullness. Therefore, for the first time, a systematic review on the effects of ACT on sleep quality has been conducted.

**Methods:**

This systematic review was performed to determine the effect of ACT on insomnia and sleep quality. To collect articles, the PubMed, Web of Science (WOS), Cochrane library, Embase, Scopus, Science Direct, ProQuest, Mag Iran, Irandoc, and Google Scholar databases were searched, without a lower time-limit, and until April 2020.

**Results:**

Related articles were derived from 9 research repositories, with no lower time-limit and until April 2020. After assessing 1409 collected studies, 278 repetitive studies were excluded. Moreover, following the primary and secondary evaluations of the remaining articles, 1112 other studies were removed, and finally a total of 19 intervention studies were included in the systematic review process. Within the remaining articles, a sample of 1577 people had been assessed for insomnia and sleep quality.

**Conclusion:**

The results of this study indicate that ACT has a significant effect on primary and comorbid insomnia and sleep quality, and therefore, it can be used as an appropriate treatment method to control and improve insomnia.

## Background

Sleep is known as a complex, active and repetitive physiological and behavioral phenomenon. During sleep, a person’s perceptual detachment from the environment and a lack of response to it are observed [1]. Sleep can be measured in terms of quality and duration, and these two parameters have little overlap. In general, sleep quality is a subjective index that measures how a person experiences sleep, while sleep duration is simply the measurement of the length of time a person sleeps. Disruption to any of these parameters leads to insufficient sleep and increases drowsiness during the day [2].

Insomnia is a 24-h disorder that occurs throughout the day and night [3]. It is known as the most common type of sleep disorder and can be instigated by primary causes or can be comorbid (due to another illness) [4]. There are different reasons for insomnia; it can also be developed occasionally, repeatedly and continuously. According to the International Classification of Sleep Disorders-Third Edition (ICSD3), insomnia is a condition in which there are sleep initiation or maintenance problems, adequate opportunities and cicumstances to sleep, and daytime consequences. If a person encounters these conditions 3 or more times during a week and the condition persists for 3 months, s/he will be diagnosed with insomnia [5]. The exact type of insomnia can be detected by polysomnography [6]. During insomnia, the cerebral cortex is more active, which increases hyper arousal in the individual [3]. Insomnia is a serious mental disorder and affects the quality of life, increasing fatigue during the day and the occurrence of various cardiovascular diseases and diabetes [7, 8]. Despite the high prevalence, there is still little known about this disorder [7].

Cognitive Behavioral Therapy is the first-line treatment for chronic insomnia [9]; This behavioral therapy is designed based on the operationally defined learning theory and its adaptation to empirical models. Behavioral therapy has three types: traditional, cognitive behavioral, and the third-wave thereapies [10]. The cognitive-behavioral therapy was introduced by using traditional behavioral therapy principles and special emphasis on cognitive factors and processes [11].

CBT-I is a multi-component approach, in which components such as psychology education in sleep health, behavioural interventions such as stimulus control and cognitive techniques are adopted [12]. CBT-I affects the factors influencing insomnia preservation. These factors include dysregulation of sleep drive, sleep-interfering behaviours, and cognitions [13].

CBT-I in the short term has the same effect as drug therapy on insomnia. Since the drug treatments pose long-term side effects in people with insomnia, the desire to treat with CBT has increased [14, 15]. A meta-analysis study by Geiger-Brown et al. (2015) found that sleep quality improves after the CBT-I treatment. This improvement was more pronounced 3 to 18 months after the treatment [16]. According to another meta-analysis conducted by van Straten et al. (2018), the effect of CBT treatment on insomnia intensity indicators, sleep quality, sleep efficiency and delayed onset of sleep was statistically significant, which can be concluded that this method is effective in treating insomnia [17].

**Fig. 1:**
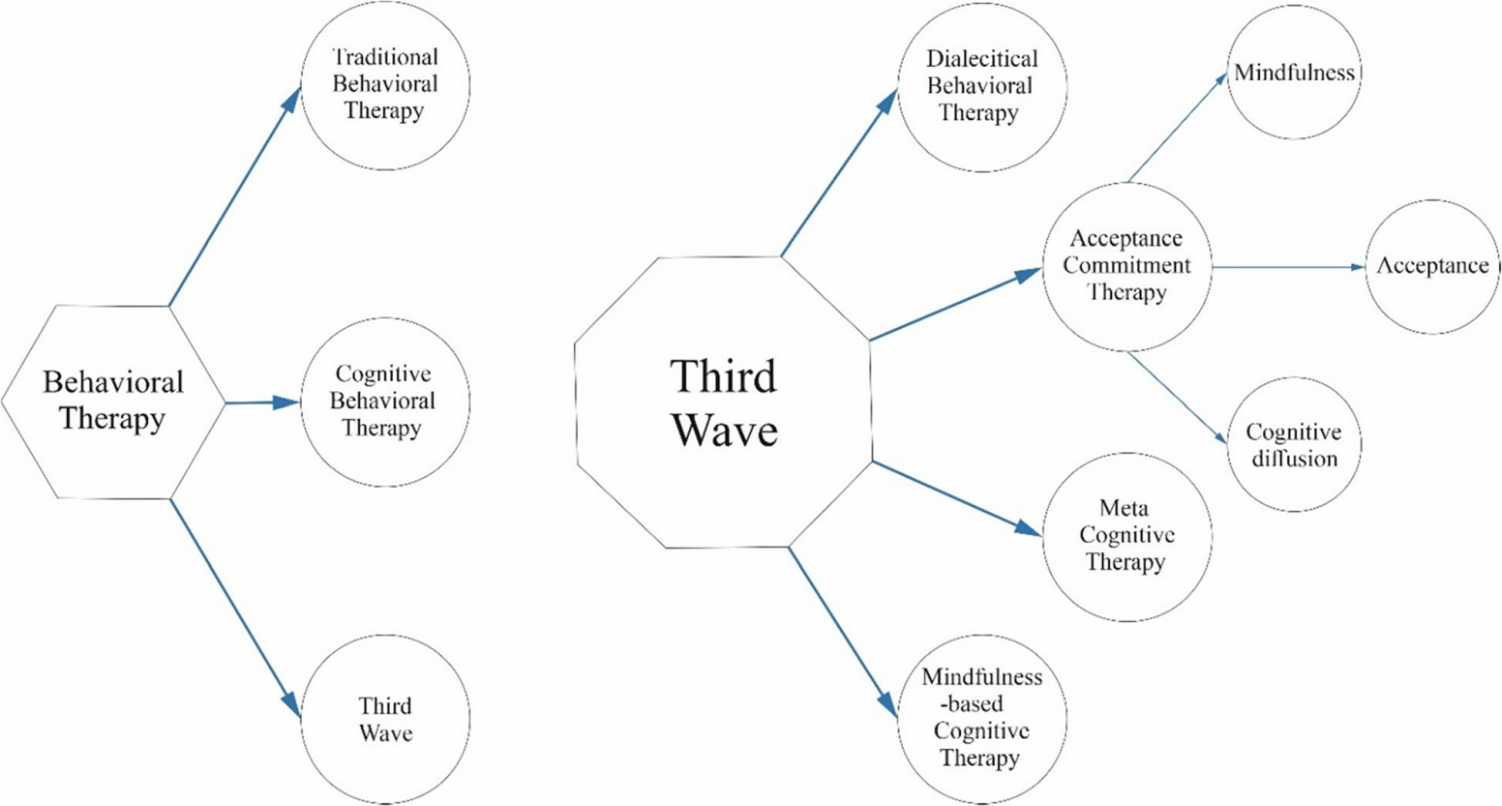
Types of Behavioral Therapy Methods and the basis for Acceptance and Commitement Therapy

Acceptance and commitment therapy (ACT) is known as one of the third-wave behavioral therapies. The third wave approaches, in addition to emphasizing the form of psychological phenomena, also have a special emphasis on the functions and contents. Third-wave interventions use concepts such as mindfulness, acceptance, and cognitive diffusion to alter people’s performance and relationship to events. Whereas in previous generations of behavioral therapies, only the direct change in events was considered [10]. ACT is known as a psychological intervention based on modern behavioral psychology in which individuals change their relationships with physical thoughts and feelings [18, 19]. In fact, ACT is based on a comprehensive scientific philosophy called functional contextualism, in which functional verbal and behavioral hypotheses are reflected in ACT in several ways [20]. ACT includes 6 treatment processes: Acceptance, Diffusion, Contact with the percent movement, Self as context, Values, and Committed action [19]. Moreover, instead of making changes to the form of experience, therapists amend its functions (Fig. 1) [20].

Several studies have reported the effect of ACT on various mental disorders, however, only a few research works have focused on the effect of ACT on insomnia. For instance, studies have examined the effect of mindfulness as one of the third wave interventions, as well as one aspect of ACT that has been shown to improve mindfulness for sleep quality improvement [21]. A case example research work by Lunde & Norhus (2009), on a 70-year-old person, found that ACT improves sleep quality [22]. Since sleep disorder is known to be one of the most common disorders in the world, and if left untreated, it will add further pressure on society, its treatment is vital. Due to the side effects of long-term use of drug therapies, alternative psychological therapies can be used [23]. This systematic review examines the effects of acceptance and commitment therapy on (1) primary insomnia disorder, (2) secondary insomnia disorder, and (3) sleep quality and other sleep parameters.

## Methods

### Studies’ eligibility criteria

In this work, in order to follow a structured approach, the 4-step PRISMA meta-analysis guidelines were followed [24]. Criteria for entering the study included, (1) intervention studies, (2) studies in which the intervention was based on acceptance and commitment therapy, (3) studies in which the full text was available, and (4) studies that were assessed as high and medium quality (i.e. studies with quality score of 18 or above). The exclusion criteria are also listed in Table 1.

**Table 1:** A) Inclusion criteria, B) Exclusion criteria and C) Search Strategy in PubMed

A) 1) Intervention study 2) The studies in which the intervention was performed were based on acceptance and commitment therapy 3) Studies with full text 4) High quality studies and scores above 18	B) 1) Observational study 2) Case report 3) Case series 4) Studies whose full text was not available 5) Studies with a quality score of less than 20 6) Studies whose intervention is another method of behavioral therapy 7) Studies with distorted data
**C)** (acceptance commitment therapy[mesh] OR Acceptance based[tiab] OR accept* commit*[tiab]) AND (insomnia[tiab] OR sleep wake disorder[mesh] OR sleep[tiab] OR “sleep quality”[tiab] OR sleep problem[tiab] OR sleep parameters[text word] OR objective sleep[text word] OR sleep maintenance[text word])	

### Search strategy and method

This systematic review was performed to determine the effect of ACT on insomnia and sleep quality. To collect articles, the PubMed, Web of Science (WoS), Cochrane library, Embase, Scopus, Science Direct, ProQuest, Mag Iran, Irandoc, and Google Scholar databases were searched, without a lower time-limit, and until April 2020. The keywords that were used were Acceptance commitment therapy, Acceptance based, accept* commit*, insomnia, sleep wake disorder, sleep, “sleep quality”, sleep problem, sleep parameters, objective sleep, sleep maintenance, and the process followed the search approach required for each database; For instance, Table 1. presents the search strategy used for the PubMed database, and lists the inclusion and exclusion criteria.

In order to examine the gray literature (dissertations, conference proceedings), the assessment of other related sites was also conducted. However, due to the lack of proper referencing mechanism, the very low quality of the results presentation in some of such articles, and also due to resources limitations, the authors removed the gray literature from this work.

In order to maximize the comprehensiveness of the search, the lists of references used in all related articles found in the above search were manually reviewed. Initially, articles that were repeated in various databases were removed from the selection. Then, a list of the titles of all the remaining articles was prepared, to evaluate the articles in a structured way. At the first stage, i.e. screening, the title and abstract of the articles were carefully examined, and considering the inclusion and exclusion criteria, unrelated articles were removed. In the second stage, i.e. eligibility assessment, the full texts of the possible related articles remaining from the screening stage were examined, based on the inclusion and exclusion criteria, and similarly the ineligible articles were excluded. To prevent subjectivity, all stages of resource review and data extraction were conducted by two reviewers independently. If an article was not included, the reason for the exclusion was mentioned. In cases where there was a disagreement between the two reviewers, a third person reviewed the article.

### Quality assessment

In order to evaluate the quality of articles (i.e. methodological validity and results), a checklist appropriate to the type of study was used. CONSORT checklists are commonly used in interventional studies to critique and evaluate quality of articles [25]. The CONSORT checklist consists of six scales/general sections including: title, abstract, introduction, methods, results, and discussion. Some of these scales have subscales, resulting in a total of 37 fields. In fact, these 37 fields represent different methodological aspects of a piece of research, such as title, problem statement, study objectives, study type, statistical population, sampling method, definition of variables and procedures, data collection methods, statistical analysis methods and findings. The maximum score that can be obtained from CONSORT is 37; Considering the score of 18 as the cut-off point [26], articles with scores of 18 or above were considered as medium or high-quality articles. Articles with a score below 18 were considered as low quality research works with respect to their methodological framework. In this study, low quality articles were excluded from the final selection.

## Results

### Article selection

**Fig. 2:**
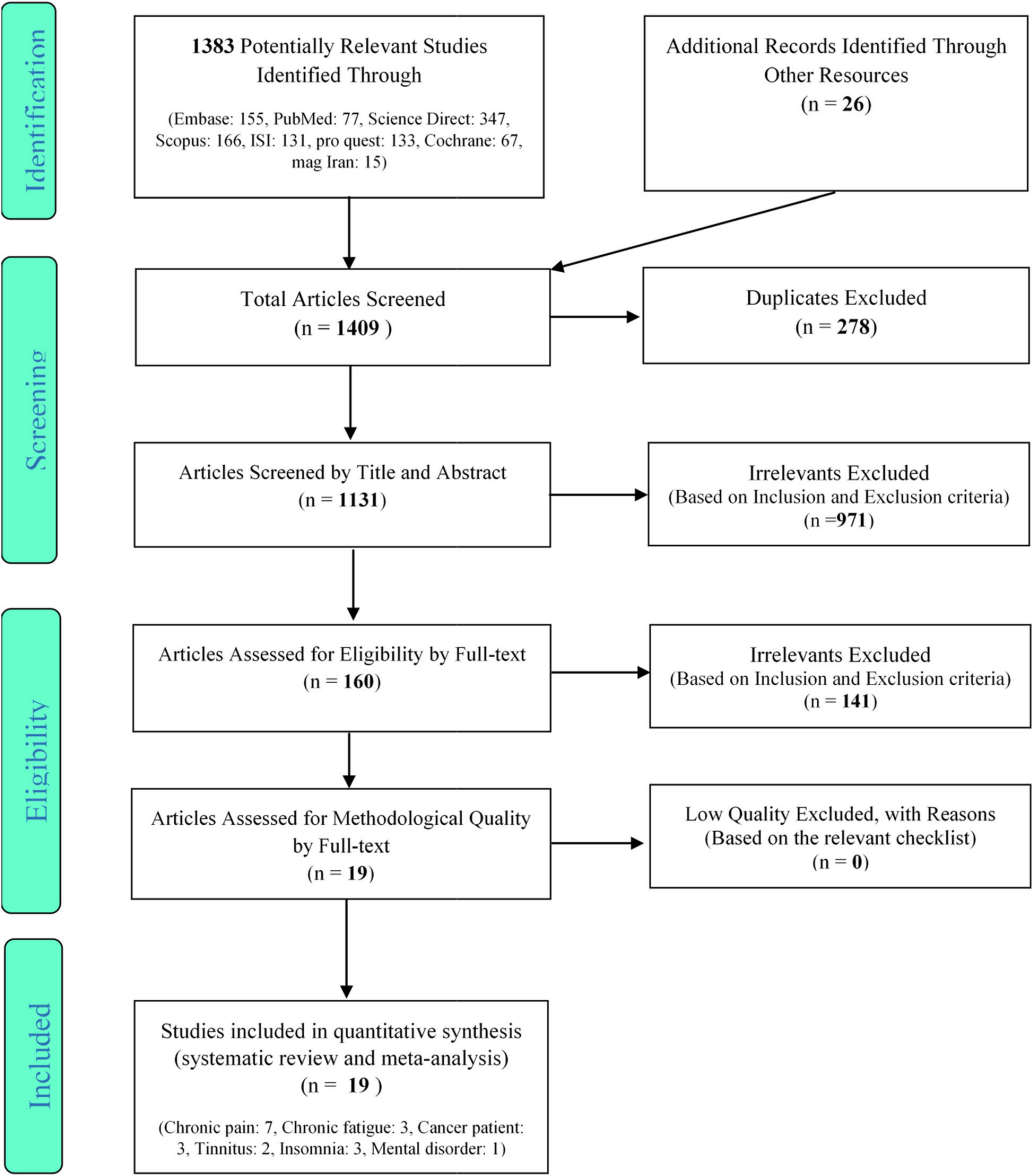
PRISMA flow diagram demonstrating the stages for inclusion of studies in the systematic review

A total of 1409 articles were collected from various databases. After removing 288 duplicate articles, the initial evaluation stage was conducted in accordance to the inclusion and exclusion criteria. At this stage of the systematic review process, by reviewing the title and abstract of 1131 articles, 160 articles were approved and entered the secondary evaluation phase. After reviewing the full text of the articles based on the inclusion and exclusion criteria, and also assessing the quality of the articles using the CONSORT checklist [26], 19 articles entered this systematic review study; Fig. 2.

### Articles quality

After the implementation of the systematic review process, 19 intervention studies were approved and evaluated. These studies are described in Table 2. In general, Randomised Control Trial (RCT) studies were of better quality, which could be due to the high risk of publication bias among them. The methodological quality of the articles included in this study was determined using the CONSORT checlist, which offered a score ranging from 18 to 28, with an overall average score of circa 22. In 8 studies, the included data were redacted [26]. In a small number of articles, using linear mixed effect models, the parameters were generated by taking into account the missing data, indicating intention-to-treat analyses.

**Table 2:** Demography details of the studies

Rows	Name and year	country	Study type	population	No. of participants intervention group, Mean age (SD), Female percent	Type of intervention, Mode of delivery, Sessions duration	No. of participants comparator, Mean age (SD), Female percent	Measures taken in the comparison group	Measurement criteria and tools	Follow up duration	Summary of outcome	Quality rate of study
1	Clarke, S. P. 2017 [27]	England	Pilot randomized controlled trial	Hip and knee osteoarthritis	16, 66 (7.3), 75%	ACT, Groups of 4 to 6 people, 6 sessions 90 min per sessions, 6 week	15, 67 (10.7),	none	Sleep and well-being (ICOAP)	2 and 4 month follow up	The average score of sleep and well-being decreased slightly after two months of follow-up, which was not statistically significant. This score decreased again after a 4-month follow-up period, which was statistically significant. During this time, there was no change in the mean score of the control group.	26
2	Craner, J. R. 2020 [28]	America	Treatment outcome study	Chronic pain	137, 48.84 (16.31), 78.8%	ACT+ other treatment, 10 sessions, 2–4 h, Almost 10 week,2 or 3 day per week	–	–	Insomnia (ISI)	–	The average score of insomnia decreased after treatment, and it was found that this treatment had a significant effect on improving insomnia, which was also statistically significant.	19
3	Farhang, Maryam .2017 [29]	India	quasi-experimental clinical trial	Mental disorder patient	11, 33.54 (8.92), All women	ACT, 8 sessions, 75 min per session, one month	11, 32.45 (11.3), All women	Normal drug treatment	Anxiety and insomnia (GHQ)	–	The average score of anxiety and insomnia decreased during treatment which shows improvments; This decrease was statistically significant. The score of the control group did not change significantly.	18
4	Herbert, M. S. 2017 [30]	America	Randomized Non-Inferiority clinical Trial	Chronic pain	128, 50 (13), 18% In person: 65 individual, Video-teleconference:63 individual	ACT and normal pain tratment, In person, Video-teleconference, 8 session, 60 min 8 week,	–	–	Sleep quality (PSQI)	3 and 6 month follow up	The mean score of sleep quality changed slightly and was not observed in any of the modified intervention methods. The observed small changes were not statistically significant.	28
5	Hesser, Hugo.2012 [26]	Sweden	Randomized contorted trial	Tinnitus	35, 50.1 (16.4), 49.2%	ACT self-help internet delivered (online ACT), 8 session, 120 min 8 week	1:Control group(32), 48.4 (14.2), 43.8% 2:CBT(32) 48.8 (13.4) 43.8%	1: None 2:CBT self-help internet delivered, Face to face, 8 session, 120 min 8 week	Insomnia (insomnia severity index)	1 year follow up	The mean score of insomnia was reduced equally by CBT and ACT. However, follow-up after one year showed that the severity of insomnia in the ACT treatment method was almost higher than the initial state and the sleep intensity in CBT was almost back to normal. The control group also showed a slight decrease in the severity of insomnia during the 2 months of treatment.	25
6	Jacobsen, Henrik Børsting. 2017 [31]	Norway	pre-post design	Chronic fatigue	140, 43.9 (9), 80.7%	ACT, Groups and individual 8sessions, 150 min, 17 days	–	–	Insomnia (ISI)	–	A decrease in the mean score of the insomnia severity after treatment was observed, which was statistically significant.	21
7	Kallestad, H. 2015 [32]	Norway	Trial, repeated measures treatment	Chronic fatigue	122, 44 (8.9), 80.3%	ACT, Groups and individual 7sessions, 17 days,	–	–	Insomnia (ISI)	–	The mean score of insomnia decreased significantly after the intervention, which was statistically significant.	19
8	Khazaie, H. 2019 [21]	Iran	quasi-experimental clinical trial	Chronic Insomnia	12, 40.5 (8.36), 58.3%	ACT, Individual 8 sessions, 60 min, 8 week	–	–	Sleep quality (PSQI)	3 month follow up	The average score of sleep quality after treatment and following a 3-month follow-up was decreased, which indicates the positive effect of ACT on sleep qauality.	20
9	Lang, A. J. 2017 [33]	America	Randomized controlled trial	Veterans with chronic pain	80, 34.5 (7.9), 19.3%	ACT, individual sessions, 12 sessions, 60 min	80, 34.0 (8.1), 21.2%	present-centered therapy (PCT), 12sessions, 60 min,	Insomnia (ISI)	3,6,9 and 12 month follow up	The mean score for insomnia severity was decreased significantly, after ACT treatment, and this was statistically significant. However, PCT did not have such an effect on improving insomnia.	26
10	Mosher, C. E. 2019 [34]	America	pilot randomized trial examined	Lung cancer AND caregivers	50, 63.20 (11.27), 60%	ACT telephone based, 6 sessions, 50 min, 6 week	50, 62 (13.13), 64%	Education/Support, other similar psychological intervention, supportive listening and directing, 6 sessions	Sleep disturbance (PROMIS)4 Item	–	The mean score of insomnia did not change much following the intervention. The analysis of the Group X time effect did not show such a change. Moreover, the effect of other psychological interventions on sleep disorder was reported higher than ACT.	26
11	Mosher, C. E. 2018 [35]	America	pilot randomized trial examined	Metastatic breast cancer	23, 59.30 (11.95), All women [19]	ACT telephone based, 6sessions, 50–60 Minutes, 6 week.	24, 53.29 (10.93),	Education/Support, 6 sessions All women	Sleep-related impairment (PROMIS)8 item, Sleep disturbance (PROMIS) 4 item	8 AND 12 week follow up	The ACT intrvention group showed little improvement in sleeplessness after 8 and 12 weeks of follow-up, however, this was not statistically significant.	27
12	Päivi, Lappalainen.2019 [36]	Finland	randomized controlled trial	insomnia	43, 56.05 (11.05), 74.4%	ACT Internet-delivered self-help, 6 sessions, 6 week	40, 50.78 (15.26), 52.5%	Control	Sleep quality (BNSQ), Insomnia (ISI), Sleeping difficulties (ESS), Recognize insomnia from normal sleep (DBAS)	6 month follow up	The intervention had a positive and significant effect on improving sleep disorder, and sleep quality in patients with chronic insomnia.	25
13	Simister, H. D. 2018 [37]	Canada	randomized controlled trial	Fibromyalgia	34	(online ACT) + treatment as usual (TAU), 7 sessions 2 month	33	TAU,	Sleep quality (PSQI)	3 month follow up	ACT was effective in improving sleep quality during treatment, however decreased during follow-up. This effect was not statistically significant.	26
14	Vethe, Daniel. 2018 [38]	Norway	randomized controlled trial	Chronic fatigue	89, 61 (9), 85.4%	ACT, Individual and group, 7 h per day, 17 days	–	–	Insomnia (ISI)	12 month follow up	The mean score of insomnia decreased significantly during the follow-up period, which was statistically significant.	25
15	Wells-Di Gregorio, S. M. 2019 [39]	America	Pilot randomized controlled trial	Advanced cancer	17, 55.59 (7.25), 76%	CBT-ACT, face to face and video session, Two person and individual 3 sessions, 90 min, 6 week	11, 58.0 (9.35), 91%	TAU	Insomnia (ISI), sleep diary (SOL), (WASO (TST),	–	Improvement of sleep quality, sleep delay, ISI severity were significantly different between the intervention and control groups, from the beginning up to week 6.	24
16	Westin, V. Z. 2011 [40]	Sweden	randomized controlled trial	tinnitus	20, 53.5 (12.84), 64%	ACT, Individual, On average 8.37 sessions per person and maximum of 10 sessions,	1)20, 48.95 (14.5), 40% 2)22, 49.59 (11.86), 36%	1) tinnitus treatment therapy (TRT) 2)control	Insomnia (ISI)	6 month follow up	Mean score for insomnia severity in the ACT intervention group decreased after treatment over 6-month, 18-month follow-up periods, and this was statistically significant. TRT intervention did not have a positive effect on improving sleep quality.	25
17	Wiklund, T. 2018 [41]	Sweden	randomized controlled trial	chronic pain	81	ACT-bsm, Groups, 7 session, 120 Minutes, 8 week	1)78, 2)73	1)Exercise 2) control	Insomnia (ISI)	6 and 12 month follow up	The average insomnia score in ACT intervention decreased after treatment and the 6 months follow-up, which was not statistically significant. The 12-month follow-up also showed a decrease in the insomnia, which was statistically significant. The effect of exercise on improving sleep quality was also evident, which was significant.	25
18	Zakiei, A. 2019 [42]	Iran	Single-arm Trial Plan	Insomnia	4, 38.5 (10.37) 50%	ACT, 8 sessions, Individual,8 week	–	–	DBAS, SPA, PSQI, Sleep diary (TST, SOL, SE)	3 month follow up	ACT improves sleep quality in people with insomnia. This situation was also observed during the follow-ups.	20
19	Zetterqvist, V. 2018 [43]	Sweden	clinical pilot study	Chronic pain	16, 38.19 (14.13), 68.8%	ACT, Group and sometimes individual 6 sessions and 1 sessions in the follow up, 120 min,	–	–	Insomnia (ISI), Sleep diary((SE), (SOL), (WASO), (TST))	3 month follow up	In general, the intervention has a direct effect on improving the insomnia severity and other parameters related to sleep quality.	23

### Participants

The sample size in the articles included in this study ranged from 4 to 232. A total of 1577 people were assessed for primary and secondary insomnia and sleep quality, of which 1058 were in the ACT intervention group. In the comparison or control groups, 32 people were treated with CBT (cognitive behavioral therapy), 80 people were treated with present-centered therapy, 20 people were treated with Tinnitus Retraining Therapy, and 78 people were treated with exercise therapy. The type of intervention was unclear for 129 patients, and no treatment was performed on 180 people. A total of 11 out of the 19 studies had a control or comparison group. All participants in the studies were all adults i.e. over 18 years old. Most of the participants were women, which potentially shows that women suffer from insomnia more than men.

In a research conducted by Hesser et al. [28], in Sweden, there were 35 participants in the intervention group and 32 participants in the comparator group, with the CBT measurements conducted using an internet-based self-administered questionnaire. In this work, the mean score of insomnia was reduced equally by CBT and ACT. However, follow-up studies after a year showed that the severity of insomnia in the ACT treatment method was higher than the initial state and the sleep intensity in CBT was almost back to normal. The control group also showed a slight decrease in the severity of insomnia during the 2 months of treatment.

Nonetheless, in patients who do not respond well to CBT-I treatment, ACT is recognised as an alternative treatment [13].

In most studies, the effect of ACT was measured on various diseases. In other words, most studies have examined the effect of ACT on comorbid insomnia. Considering Tables 2, 5 studies have assessed the effect of ACT on people with chronic pain [30, 33, 37, 41, 43]. One research work also studied the Fibromyalgia condition which has symptoms such as widespread pain and fatigue [27]. In another work, patients with Osteoarthritis who also suffered from chronic pain were examined [31]. The effects of ACT on various aspects of chronic fatigue, especially sleep, were investigated in three pieces of research [32, 34, 38]. Three studies also examined patients with cancer [35, 39, 40]. Two articles studies patients with Tinnitus [28, 29] and another work examined people with mental disorders [36]. Only 3 research works have examined the effect of ACT interventions on primary insomnia [21, 42, 44].

Different criteria were used to measure insomnia and sleep quality in the selected research works. Some of these works have used several methods to measure sleep patterns in their samples. Overall, in 11 studies, insomnia was measured by the Insomnia Severity Index (ISI) [28–30, 32, 34, 37, 38, 40–43]. In 4 studies, sleep quality was assessed using the PSQI questionnaire [21, 27, 33, 44]. In another work, parts of the ICOAP questionnaire examined sleep and well-being in the study sample [31]. Another piece of research utilised a GHQ questionnaire to measure anxiety and insomnia [36]. Sleep quality was measured in an article with the BNSQ questionnaire [42]. In 2 works, using 4 items from the PROMIS questionnaire, sleep disturbance was evaluated [35, 39]. Furthermore, in a study, sleep related impairment was measured by the PROMIS questionnaire [39]. The ESS questionnaire, which measures daytime sleepiness, was used in 1 research work, and the DBAS questionnaire, was used in 2 studies [42, 44]. The sleep diary criteria, which includes sleep onset latency (SOL), wake time after sleep onset (WASO) and total sleep time (TST), were examined in 2 articles [34, 37]. Sleep efficacy (SE) was also evaluated in another work [37]; (Table 2).

### Investigating the interventions

In the selected studies, the intervention was performed in different ways. In 4 studies, the protocol therapy was performed in person, and individually on the study sample [21, 29, 41, 44]. In 2 other studies, all sessions were performed in groups [31, 43]. In one of these research works, acceptance and commitment therapy based stress management (ACT-bsm) was utilised [43]. Some other studies used both group and individual methods to hold meetings [32, 34, 37, 38]. The telephone based ACT method was reported in 2 articles [35, 39]. ACT was performed virtually and in an online platform in 3 other works [27, 28, 42]. In another piece of research, face-to-face and video conferencing were performed simultaneously with two different groups [33]. In another research project, face-to-face and virtual video-based sessions were scheduled, in which the combined CBT-ACT therapy was performed [40].

The number of treatment sessions reported in the examined research works also varied. In general, ACT was presented with different protocols in different projects, which led to variances in the number of study sessions. In 6 projects, ACT was presented in 8 sessions [21, 28, 32, 33, 36, 44]. The average number of sessions presented in the study by Westin et al. (2011) was 8.37. In this work, a maximum of 10 sessions were planned to present the content [29]. Four other studies scheduled 7 sessions as part of the project. In the research project conducted by Zetterqvist et al. (2018), the last session was performed when people were called in for a follow up [27, 37, 38, 43]. Some other studies used 6 sessions of therapy to present their findings in accordance with the ACT principles [31, 35, 39, 42]. The use of 3 sessions [40], 10 sessions [30] and 12 treatment sessions was also observed in the various pieces of research [41]. The timing of each session was also different. Considering Table 3, the time for presenting sessions in different studies was reported from 50 min to 7 h per day (Tables 3, 4).

**Table 3:** Investigating Changes in Sleep Intensity Questionnaire

Name	intervention /control group	Assesment	Number of sessions, time for presenting sessions	pretreatment	post treatment	month follow up, mean square	month follow up, mean square	p- value	T test / F	effect size
Craner, J. R	ACT	ISI (Insonia severity index)	10, 2–4 h	13.86 (6.89)	9.39 (5.97)	_	_	< 0.001	t = 8.56	d = 0.73
Kallestad, H.	ACT	ISI	7	12 (6.07)	9.51 (6.12)	_	_	<.001	t = 5.55	d = 0.41
Lang, A. J.	ACT	ISI	12, 60 min	15.2 (6.6)	11.8 (7.7)	_	_	< 0.05	_	d = 0.63
Vethe, Daniel	ACT	ISI	–	12.25 (5.92)	9.03 (5.92)	_	_	< 0.001	t = 5.8	d = 0.54
Wells-Di Gregorio, S. M.	ACT	ISI	3, 90 min	5.71 (0.41)	3.39 (0.39)	_	_	0.0047	_	d = −1.18
Hugo Hesser	ACT	ISI	8, 120 min	13.23 (5.80)	8.48 (5.43)	12 month, 17.32 (9.85)	_	_	_	_
	Control	ISI	–	13.78 (6.54)	11.22 (6.97)	_	_	0.1	t < 1.70	d = 0.41
	CBT	ISI	8, 120 min	14.66 (6.30)	9.93 (6.85)	12 month, 12.03 (8.39)	_	0.043	t = 2.06	d = 0.52
Westin, V. Z.	ACT	ISI	10	11.90 (4.66)	9.25 (5.17)	6 month, (9.19 (6.07))	18 month, 8.90 (5.49)	_	_	_
	Control	ISI	–	11.91 (6.60)	11.80 (6.14)	_	_	0.022	f = 5.67	d = 0.22
	TRT	ISI	–	12.60 (5.70)	13.06 (5.63)	6 month, 11.47 (5.81)	18 month, 12.57 (6.33)	0.043	f = 4.19	d (6 month) = 0.71, d(18 month) = 0.63
Wiklund, T.	ACT	ISI	7120 min	14.23 (6.00)	13.25 (6.30)	6 month, 13.24 (6.38)	12 month, 12.22 (6.38)	Post treatment = 0.071, 6 month follow up = 0.215, 12 month follow up = 0.009	_	d(Post treatment) = − 0.207, d(6 month follow up) = 0.036, d(12 month follow up) = − 0.279
	Control	ISI	–	12.76 (7.24)	13.15 (7.46)	6 month, 11.53 (7.29)	12 month, 12.59 (7.13)	Post treatment = 0.234, 6 month follow up = 0.122, 12 month follow up = 0.851	_	_
	exercise therapy	ISI	7	13.48 (6.52)	12.14 (6.55)	6 month, 11.64 (7.15)	12 month, 11.19 (6.27)	Post treatment = 0.020, 6 month follow up = 0.002, 12 month follow up = 0.001	_	d(Post treatment) = − 0.262, d(6 month follow up) = − 0.092, d(12 month follow up) = − 0.321
Jacobsen,H.B	ACT	ISI	8150 min	12.2 (6.1)	9.8 (6.4)	_	_	< 0.001	5.53	g = 0.38
Zetterqvist, V.	ACT	ISI	7120 min	20.19 (3.78)	10.75 (5.23)	3 month, 11.20 (6.71)	_			g pre-post = 2.02 (0.9,3.14), g pre-follow up =1.69 (0.59,2.78)

**Table 4:** Statistical data by evaluation criteria

Name	intervention / control	parameters	Number of sessions, time for presenting sessions	pre treatment	post treatment	month follow up, mean square	statistical analysis	df	p	F / W /β(SE)	effect size / difference in change between groups
Herbert, M. S	ACT /in person	PSQI	8, 60 min	12.48 (0.49)	11.58 (0.52)	6 month, 11.78 (0.55)	Samples T test	_	_	_	difference in change pre-post, −0.21(− 1.63_1.20), difference in change pre-follow up, − 0.14(− 1.69_1.42)
	ACT/ tele health	PSQI	8, 60 min	12.16 (0.50)	11.47 (0.61)	6 month, 11.59 (0.67)		_	_	_	
Khazaie, H.	ACT	PSQI	8, 60 min	15.58 (2.31)	5.17 (2.88)	3 month, 3.17 (2.03)	Analysis of Variance (ANOVA)	1	0.001	F = 433.77	_
Simister, H. D	ACT+TAU	PSQI	7	12.67 (3.80)	10.24 (3.60)	10.70 (4.71)	Linear mixed effects modeling (LMM)	df1 = 2	0.055	_	d(pre-post) =0.79 (0.26–1.31)
	TAU	PSQI	7	13.26 (3.80)	13.00 (3.47)	_				_	d(pre-follow up) = 0.53 (0.2–1.04)
Zakiei, A.	ACT	PSQI	8, 60 min	16 ± 2.44	5 ± 4	5 ± 4.08	Analysis of Variance (ANOVA)	_	< 0.01	_	Cohen’s d = 1.733
Päivi, L	ACT	ESS	6	6.85 (4.59)	5.67 (3.81)	5.20 (3.22)	Wald test	df = 1	0.001	W = 6.71	Cohen’s d (pre-post) = 0.41, Cohen’s (pre-follow up) = 0.69
	control	ESS	–	7.60 (4.28)	7.30 (5.09)	_		df = 2	< 0.001	W = 32.23	
Wells-Di Gregorio, S. M.	ACT	ESS	3, 90 min	8.82 (1.11)	7.99 (1.24)	_	linear mixed models	_	0.66	_	_
	control	ESS	–	8.09 (1.38)	7.91 (1.46)	_		_	0.66	_	_
Päivi, L	ACT	DBAS	6	96.33 (22.81)	86.57 (25.74)	77.47 (29.70)	Wald test	df = 1	0.001	W = 10.41	Cohen’s d (pre-post) = 0.53
	control		–	87.93 (20.62)	89.55 (18.85)	_		df = 2	< 0.001	W = 34.86	Cohen’s (pre-follow up) = 0.71
Zakiei, A.	ACT	DBAS	8, 60 min	92.75 ± 5.85	34.25 ± 16.64	3 month, 34.25 ± 14.63	Analysis of Variance (ANOVA)	_	< 0.05	_	Cohen’s d = 1.781
Mosher, C. E(2019)	ACT	PROMIS (sleep disturbance)	6, 50 mintes	10.56 (0.71)	10.24 (0.78)	10.04 (0.78)	Linear mixed effeect model	74	0.69	F Value = 0.38	_
	education support	PROMIS	–	11.24 (0.71)	10.15 (0.81)	9.85 (0.81)		ـ	ـ	ـ	ـ
Mosher, C. E(2018)	ACT	PROMIS (Sleep-disturbance)	6, 50–60 min	13.39 (3.24)	12.86 (3.06)	12 (3.27)	Linear mixed effeect model	ـ	0.14	β = − 0.49 (0.33)	d(pre-post) = −0.14
	education support	PROMIS	–	11.5 (2.67)	10.96 (2.54)	9.92 (3.23)		ـ	ـ	ـ	d(pre-follow up) = −0.43
Mosher, C. E(2018)	ACT	PROMIS (sleep related impairment)	6, 50–60 min	23.04 (7.42)	21.61 (5.24)	21.04 (6.55)	Linear mixed effeect model	ـ	0.88	β = 0.11 (0.72)	d(pre-post) = − 0.27
	education support	PROMIS	–	18.79 (6.35)	18.79 (4.51)	19.12 (4.97)		ـ	ـ	ـ	d(pre-follow up) = −0.31
Clarke, S. P.	ACT	ICOAP	6, 90 min	5 (3–7)	4 (3–6)	3 (2–4)	Mann-Whitney’s U test	ـ	P (pre-post) = 0.05	ـ	ـ
	control	ICOAP	–	6 (4–7)	6 (5–8)	6 (5–7)			P (pre-follow up) = 0.002		ـ
Farhang, Maryam	ACT	GHQ	8, 75 min	12.27 (3.35)	7 (2.41)	_	multivariate covariance analysis statistical test	1	0.001	F (pre) = 92.988	effect (pre) = 0.830
	control	GHQ	–	12.82 (4.64)	12.73 (3.95)	_		ـ	ـ	F (post) = 81.154	effect (post) = 0.810
Päivi, L	ACT	BNSQ	6	21.77 (4.45)	19.91 (5.25)	18.35 (5.42)	Wald test	df = 1	0.001	W = 6.71	Cohen’s d (pre-post) = 0.42
	control	BNSQ	–	21.65 (4.07)	21.57 (4.5)	_		df = 2	< 0.001	W = 32.23	Cohen’s (pre-follow up) = 0.69
**Name**	**intervention / control**	**sleep diary**	**Number of sessions, time for presenting sessions**	**pre treatment**	**post treatment**	**follow up**	**statistical analysis**	**df**	**p**	**F**	**effect size**
Wells-Di Gregorio, S. M.	ACT	SOL (min)	3, 90 min	52.16 (10.43)	30.78 (5.87)	_	linear mixed models	ـ	0.028	ـ	−0.86
Zakiei, A.	ACT	SOL	8, 60 min	1.52 (0.36)	0.96 (0.56)	_	Analysis of Variance (ANOVA)	ـ	< 0.05	ـ	2.806
Zetterqvist, V.	ACT	SOL (min)	7, 120 min	59 (0:39)	20 (0:10)	25 (0:17)	linear mixed effects models	_	_	_	g(pre-post) = 1.33(.25, 2.41), g (pre-follow up) = 1.08(−0.15, 2.32)
Wells-Di Gregorio, S. M.	ACT	SE	3, 90 min	0.80 (0.027)	0.9 (0.019)	_	linear mixed models	_	0.0062	_	d = 1.08
Zakiei, A.	ACT	SE	8, 60 min	51.31 (10.6)	79.61 (10.77)	_	Analysis of Variance (ANOVA)	_	< 0.05	_	d = 2.28
Zetterqvist, V.	ACT	SE	7, 120 min	68.8 (13.9)	85.8 (6.5)	86.8 (6.7)	linear mixed effects models	_	_	_	g(pre-post) = −1.57(−2.63,-0.47), g (pre-follow up) = −1.58(−2.90,-0.26)
Zakiei, A.	ACT	TST	8, 60 min	4:17 (1:26)	6:03 (0:82)	_	Analysis of Variance (ANOVA)	_	< 0.05	_	d = 3.37
Zetterqvist, V.	ACT	TST	7, 120 min	5:46 (1:54)	5:56 (1:17)	6:43 (1:26)	linear mixed effects models	_	_	_	g(pre-post) = −0.10(−1.07, 0.87), g (pre-follow up) = − 0.54(− 1.71, 0.63)
Zakiei, A.	ACT	number of awakenings	8, 60 min	1.82 (0.29)	1.32 (1.76)	_	Analysis of Variance (ANOVA)	_	< 0.05	_	d = 1.76
Zakiei, A.	ACT	subjective quality	8, 60 min	4.07 (1.40)	5.67 (1.83)	_	Analysis of Variance (ANOVA)	_	< 0.05	_	d = 3.054
Zetterqvist, V.	ACT	WASO	7, 120 min	0:46 (0:32)	0:23 (0:33)	0:22 (0:19)	linear mixed effects models	_	_	_	g(pre-post) = −0.69(−0.31, 1.69), g (pre-follow up) = − 0.87(− 0.33, 2.08)

Different statistical methods and analyzes were conducted to investigate the effect of the interventions. Nevertheless, considering the results of existing research works, in general, ACT has a positive effect on the severity of insomnia (ISI) and reduces its severity. The research conducted by Craner et al. (2020) showed a high positive effect of ACT on reducing insomnia, which was statistically significant, ranging from 13.86 ± 6.89 to 9.39 ± 5.97 after intervention [30]. Other studies have shown a positive and significant effect of the intervention on the severity of insomnia, with the following value ranges: 12.2 ± 6.1 to 9.8 ± 6.4 [32], 12 ± 6.07 to 9.51 ± 6.12 [38], 15.2 ± 6.6 to 11.8 ± 7.7 [41] and 12.25 ± 5.92 to 9.03 ± 5.92 [34], 5.71 ± 0.41 to 3.39 ± 0.39 [40], 11.90 ± 4.66 to 9.25 ± 5.17 [29], 14.23 ± 6.00 to 13.25 ± 6.30 after intervention [43]. In the work of Wiklund et al. (2018), a decrease in ISI score was observed throughout the study period (after treatment, 6-month follow-up, and 12-month follow-up), which was only statistically significant in the 12-month follow-up [43]. Moreover, another research work found a positive and significant effect of ACT intervention on ISI compared to other interventions [29]. In the study of Hesser et al. (2012), no statistical significance was observed in the comparison between the intervention group and the control group, before and after intervention [28].

The Pittsburgh Sleep Quality Index (PSQI) was used in 4 studies; PSQI showed improvement in sleep quality after intervention in 2 studies, which was statistically significant [21, 44], yet this was not statistically significant in the other two works [27, 33]. Sleep disturbance was measured in 2 research works using parts of the PROMIS questionnaire; the findings demonstrated only a minor change in the score from the questionnaire, and that it was not statistically significant [35, 39]. Sleep related impairment were also assessed with the PROMIS questionnaire, and the score changes were not statistically significant [39]. Sleeping difficulty was measured by ESS in 2 studies. In a research work conducted by Päivi et al. (2019), a comparison of pre-intervention and post-intervention intervals showed that the mean ESS score changed slightly, which was not statistically significant, while a comparison of pre-intervention time interval and follow-up showed changes that were statistically significant [42]. Another study found small changes in the ESS score that were not statistically significant [40].

In general, changes in DBAS score were reported to be decreasing, and statistically significant, which meant the positive effect of the intervention on the dysfunctional beliefs and attitudes before sleep criteria [42, 44]. The effect of ACT on sleep and well-being was measured by ICOAP. The comparison before and after the intervention did not show a significant relationship, while the comparison of the pre-intervention and the follow-up periods reported improvement in sleep and well-being, which is statistically significant [31]. The effect of the intervention on insomnia and anxiety was measured by the GHQ instrument, which was statistically significant [36]. Comparing the average sleep quality score using the BNSQ instrument at different time intervals showed a decrease in the mean score and improvement of the condition, which was statistically significant [42]. Changes in the SPA instrument, that assesses sleep problem acceptance, were reported to be statistically significant [44].

Sleep diary descriptive criteria were also assessed in a number of research works. The effect of intervention on sleep efficacy (SE) was reported to be positive. In fact, ACT significantly increased SE, and the effect of ACT on sleep onset latency (SOL) was also reported to be significant [37, 40, 44]. Two criteria of total sleep time (TST) and wake after sleep onset (WASO), were also measured. The effect of ACT on TST was significant, yet no significant effect was observed with WASO [37]. In a research conducted by Zakiei & Khazaei (2019), the effect of ACT on TST and the number of awake sleeps were measured, which showed a significant improvement in the status of these parameters [44].

In a study conducted by Clarke et al. (2017), statistical analysis was performed using the Mann-Whitney’s U test. It was reported that the passage of time has a positive impact on the improvement of sleep quality and well-being after intervention, so that the improvement of sleep and well-being in the first two months of follow-up was insignificant, whereas in the 4-month period, this was statistically significant [31]. In 6 research works, paired sample t-test was used. In 4 of these studies, the Cohen d effect was measured, which was statistically significant, and the effects in the studies were reported to be moderate or large [30, 34, 38, 41]. Herbert et al. (2017) also used a similar statistical analysis method, however, no statistical significance was observed in their work [33]. The paired sample t-test was also used to analyze the results in a research conducted by Jacobsen et al. (2017). In this test, Hedge g was adopted to measure the effect, which was calculated as 0.38 and was statistically significant [32].

Linear effect size model analysis was used in the study of Hesser et al. (2012), which reported a statistically insignificant comparison between ACT intervention and control group. CBT comparison compared to ACT showed d = 0.52, which was statistically significant. Interventions were not sustainable during the follow-up period [28]. In 2 separate studies, Mosher et al., analysed the data with linear effect model size, compared the (Group × time) data before and after intervention; results were not statistically significant [35, 39]. The linear effect model size was also used in a research conducted by Westin et al. (2011). The results of this study showed that comparing ACT versus Control before and after the test is statistically significant and ACT improves sleep quality. Moreover, comparing ACT intervention with TRT in follow-up periods of 6 months and 18 months showed that ACT is a better treatment than TRT and its effect is significant throughout the intervention and follow-up [29]. Wells-Di et al. (2019) followed the same analysis method, in which a positive and significant effect of intervention on sleep parameters was observed [31].

The study of Wiklund et al. (2018) also used linear effect model size analysis. In this work, the population was divided into two groups where there were completer and non-completer treatments. The calculated effect size for the group that completed the treatment was significant in the follow-up period of 18 months and was insignificant in other intervals. Moreover, after taking into account the total population (completer and non-completer), the intervention was not significant in any of the time periods [43]. Zetterqvist et al. (2018) also used linear effect model size in their work, according to which ISI and SE were significant in the lead time period after intervention and in the follow-up interval. TST criteria were also reported insignificant in the pre and post-intervention periods, yet statistically significant during follow-up. The WASO criterion was insignificant in both ranges [37].

## Discussion

This is a systematic review of the evidences and results reported within 19 studies with a total sample of 1577 patients suffering from primary and comorbid insomnia. These studies have focused on the effect of ACT intervention on insomnia and sleep quality. The included studies have been published in the 2012–2020 period, 6 of which were published in 2019 and 2020 [21, 30, 35, 40, 42, 44], and all studies were assessed as medium or high quality articles. This work is the first systematic review that focuses on the effect of ACT on insomnia. Despite the many differences in the primary cause of insomnia, most research works reported a positive and significant effect of acceptance and commitment therapy on improving insomnia and sleep quality. Due to the wide range of tools and criteria used for measuring sleep quality in the studies, there were also indications of statistically insignificant effect of ACT on these parameters. Moreover, as some of the comparative studies were conducted at different time intervals, the statistical significance or insignificance of the effect were therefore observed in different time periods.

ACT is based on identifying more efficient behaviourial elements. These elements cause a lasting change in one’s behavior, which results in the rise of happiness and fulfillment of personal goals [45]. Studies have shown that CBTI, as the second generation of behavioral therapies, plays an important role in improving quality and duration of sleep [46]; accordingly the result from the work of Edinger et al. have confirmed this through polysomnography. Such improvement may persist for up to 24 months [47]. Research has also shown that ACT, as much as CBT, can be as an effective method in treating physical and mental disorders such as chronic pain, anxiety and depression, as well as in treating insomnia [48].

In a work conducted by Castronovo et al. (2018), 258 patients completed the CBT-I treatment. Improvement in insomnia and sleep diaries were observed in these individuals. It was also reported that the effect of this treatment can be stable for up to 10 years [12]. Therefore, CBT-I can be considered as a suitable treatment for insomnia. However, Baglioni et al. (2020) - at the European Academy for Cognitive Behavioral Therapy for insomnia - reported that patients in Europe do not have a consistent access to CBT-I and that the use of this treatment has its drawbacks. Moreover, providing training on this method for the specialists is a challenging task [13].

ACT is known to treat long-term illnesses. Cancer, childhood illnesses, pain, heart disease and diabetes are some of the diseases that have been treated by this therapy. The distinctive feature of ACT is that it can greatly reduce the side effects of not taking medicine on time, however, the effectiveness of the long-term use of this treatment is still debated by researchers [49]. Other evidences suggest that ACT interventions are also used in a wide range of psychotic disorders [10]. A research conducted by Orsillo et al. (2005) argued that ACT could be identified as an effective treatment for Post-traumatic stress disorder (PTSD) [50]. In the present study, sleep quality was assessed in patients with chronic pain, cancer, and tinnitus, before and after intervention. Several research works have assessed the results of pre-treatment, up to follow-up interventions, and statistical significance of the effect of the intervention was reported in some the works [27, 29, 34, 42, 43]. According to a 2016 cohort study conducted in a population with chronic pain, ACT improved insomnia, sleep quality, and sleep efficiency during treatment and follow-up for 9 months after the intervention. This improvement was statistically significant [51].

ACT is known as one of the third wave treatments and originates from the cognitive behavioral therapy (CBT), which increases psychological flexibility. Existing research works have shown that the effect of ACT in a 3-year follow-up has also been stable [52]. The use of this behavior therapy method has been observed in a wide range of diseases, which indicates the flexibility of this method. This has also increased the desire to use ACT in recent years [49]. On the other hand, the variety in the applications of ACT methods e.g. virtual, telephone-based and face-to-face therapies, also highlights the flexibility of this treatment approach. Most of the treatment strategies used in ACT are derived from other approaches [53]. ACT pursues a pragmatic and flexible approach, and reduces inefficient efforts to control conditions and thoughts, and offers an adaptive response to diseases [54].

On the whole, ACT is performed with the aim of mental training, increasing motivation, psychological flexibility and self-efficacy in an individual [32]. Other goals, such as reducing efforts to control events, identifying personal goals, and making commitment to take action, are also followed in this treatment [28, 42]. A study by Mosher et al. (2019) showed that ACT attempts to develop of mindfulness skills; the authors went on to argue that it can reduce cancer effects in patients [35]. Moreover, where patients suffer from chronic pain, the goal is to influence the pain acceptance, increase participation in life activities, manage thoughts and feelings, change expectations and goal of a treatment, and express the possibility of living with pain and improving quality of life [30, 31, 33].

In most studies, the effect of ACT on improving insomnia and sleep quality is observed. The research projects conducted by Khazaie et al. [21] and Zakiei et al. [44] examined the effect of ACT on patients with chronic insomnia. According to the results of these studies, ACT improves the sleep quality of these patients. The results of these research works also showed that the effect of interventions is stable, e.g. the 3-month follow-up exposed that the quality of sleep is better than the time before the intervention. The study by Päivi et al. reported the 6-month stability of the effect of ACT on sleep quality [42]. The positive effect of ACT on the parameters of sleep diaries was also reported in the work of Zakiei et al. [44].

In a study by Jacobsen et al. (2017) in Norway, patients suffering from chronic fatigue were treated. In this work, ACT increased the quality of life and reduced fatigue in the patients, and improved insomnia in the study sample [32]. This result was also observed in the study of Kallestad et al. (2015). In this piece of research, patients with chronic fatigue were treated [38]. A study by Vethe et al. (2018) reported that ACT could improve insomnia in people with chronic fatigue. Additionally, the stability of the intervention was also observed in this study. ACT in the one-year follow-up period also has a positive effect on physical condition and sleep quality of patients [34]. Therefore, it can be concluded that ACT can be an effective treatment for the symptoms of chronic fatigue.

Craner et al. (2020) examined 137 patients with chronic pain. In this research work, ACT was used in 10 sessions with normal treatment methods that improved insomnia in the patients. Improvement of pain status, and increase in pain acceptance were also observed in the patients [30]. Studies by Lang et al. [41], Simister et al. [27], and Zetterqvist et al. [37] also reported improvements in insomnia among patients with chronic pain. On the other hand, studies conducted by Clarke et al. [31], and Herbert et al. [33] did not show a positive and significant effect of ACT on insomnia and sleep quality. Nevertheless, in the work of Wiklund et al. (2018), a positive and significant effect of this intervention was observed after 12 months. In other words, the effect of ACT immediately after intervention and 6 months after intervention was not statistically significant [43].

Cancer patients were also considered as a sample in 3 articles. According to a 2019 study by Mosher et al., which was performed on patients with advanced lung cancer, ACT only had small effect on improving the sleep status of patients and their companions [35]. In another research work by Mosher et al. (2018), the effect of ACT on the improvement of breast cancer patients was not observed [39]. However, Wells-Di Gregorio et al. (2019) reported the positive and significant effect of ACT in improving insomnia in cancer patients [40].

**Fig. 3:**
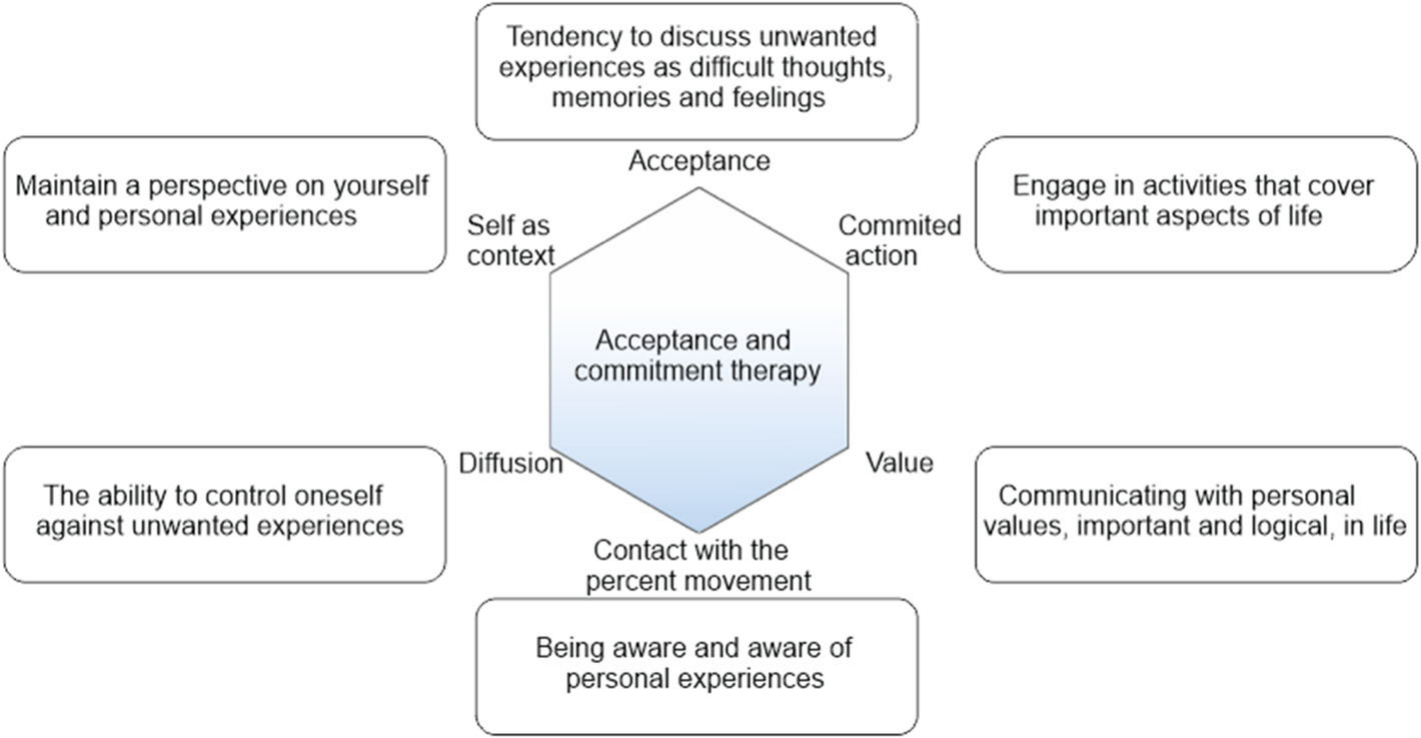
Therapeutic processes of acce,ptance and commitment therapy

Research works have demonstrated that ACT can also be effective in controlling insomnia. Since sleep is not voluntarily controlled, people may experience feelings of hopelessness, anxiety, and mood swings. This arousal can cause sleeplessness. As a person with insomnia tries to control his or her thoughts and feelings, his or her insomnia is exacerbated [46]. Clarke et al. (2017) applied the techniques to offer their patients with acceptance using the concepts and exercises of achieving the will to fight pain, limiting condition control, focusing on personal experiences, and emphasising the notion of ‘living your life’ [31]. Other studies have used the technique to focus on experience, self-awareness about positive and negative thoughts and feelings, as well as the acceptance of attitudes and unwanted thoughts (Fig. 3) [33, 36, 42].

Cognitive defusion techniques generally demonstrate that dysfunctional cognition directly plays a role in negative emotions. They are used to change the shape and frequency of undesirable behaviors and thoughts [55, 56]. In fact, these techniques reduce the negative feelings by changing the background of thoughts [57]. After reviewing various studies, it was found that teaching how to accept painful events and thoughts without changing its nature, creating creative frustration, as well as identifying unwanted thoughts and learning to describe them are among such cognitive methods [28, 33, 42, 43].

During the ACT process, contact with the present resulting moment and life is taught within the right environment, and the purpose of this work is to connect the person more with his/her surroundings. The technique itself is very important as a basis, during which the person talks about his/her experiences without being dependent on them, the acceptance of self and loss are strengthened, and in fact, the person recognises himself/herself as the source of thoughts, feelings and emotions [58]. In other literature, different techniques are used to present the concept of awareness, such as freedom of thought, expression of existing contradictions between experience and mind, training to observe inner experiences, and presence in the moment [33, 36, 37, 44]. Values have never been a static goal and are constantly changing, leading to personal growth. Using the concepts presented in the ACT protocol, a patient determines own valuable personal goals and strives to achieve them through committed and goal-based actions, which improves the quality of life [10, 56]. In relation to sleep quality, ACT increases patient’s desire to have a good sleep experience by making changes in attitudes and thoughts of the atmosphere, and attracts his/her attention to the negative and defective cycle of these thoughts with a view to improve sleep quality [21].

### Limitations

Some of the initially collected studies were in the form of dissertations and were excluded from the systematic review. The lack of access to the full text of some other articles is another limitation of this work. Due to the prevalence of the disease, variations in the samples and analysis techniques, and differences in outcomes,it was not possible to also conduct a meta-analysis of the reported results. Except for 2 studies, no polysomnography was used in other studies, which could affect the reliability of their findings.

## Conclusion

In this systematic review, the effect of ACT on insomnia and sleep quality was investigated. The results of 3 studies showed that ACT could play an effective role in improving sleep quality in patients with primary insomnia, and the results were statistically significant. Other research works have demonstrated that ACT is effective in reducing the severity of insomnia, and similarly the results were statistically significant. It can also be argued that ACT can improve sleep quality. In 2 studies, no significant relationship was observed between the treatment used and sleep quality improvement. Moreover, the stability of improvement in sleep quality was measured in different follow-up periods, which can be argued that the improvements reported in 7 studies were significant, and no significant relationship was observed in another 4 studies.

## Data Availability

Datasets are available through the corresponding author upon reasonable request.

## Abbreviations

ACT: Acceptance and Commitment Therapy
ICSD3: International Classification of Sleep Disorders-Third Edition
CBT: Cognitive behavioral therapies
CBTI: Cognitive behavioral therapy for insomnia
SOL: Sleep onset latency
WASO: Wake time after sleep onset
TST: Total sleep time
SE: Sleep efficacy
ACT-bsm: Acceptance and commitment therapy based stress management
CONSORT: Consolidated Standards of Reporting Trials
PRISMA: Preferred Reporting Items for Systematic Reviews and Meta-Analysis.
